# Associations of interpersonal trust with juvenile offending/conduct disorder, callous-unemotional traits, and criminal recidivism

**DOI:** 10.1038/s41598-022-11777-6

**Published:** 2022-05-09

**Authors:** Marcel Aebi, Melanie Haynes, Cornelia Bessler, Gregor Hasler

**Affiliations:** 1 Research and Development, Corrections and Rehabilitation, Department of Justice and Home Affairs, Canton of Zurich, Hohlstr. 552, 8090 Zurich, Switzerland; 2 Department of Forensic Psychiatry, University Hospital of Psychiatry Zurich/University of Zurich, Zurich, Switzerland; 3 Translational Research Center, University Hospital of Psychiatry and Psychotherapy Bern, Bern, Switzerland; 4 Unit of Psychiatry Research, University of Fribourg, Fribourg, Switzerland

**Keywords:** Human behaviour, Psychology, Risk factors

## Abstract

Interpersonal trust has been described as a core dimension of cooperative, mutually beneficial interpersonal relationships but it is unclear if it is related to antisocial behaviours in youth. The present study aimed at analysing a subsample of male juveniles who committed serious violent offenses and met criteria of conduct disorder (JO/CD), and a subsample of healthy controls (HC) using a series of trust games (TGs). Twenty-four male JO/CD and 24 age matched male HC performed a series of eight one-shot TGs against different unknown human respectively computer opponents. Mixed model analyses found a non-significant trend that JO/CD invested less points than HC during TGs. In the subsample of JO/CD, the overall investment in TGs was found to be negatively associated with self-reported uncaring behaviours and officially reported general re-offenses. Our findings suggest some indication of an impaired ability of JO/CD to initiate mutually trusting relationships to others that should be addressed in further research. Trust is a promising factor to predict general criminal recidivism and can be a target for treatment of juveniles who committed violent offenses, for example through the building of stable relationships to care givers. This study encourages future studies to investigate the effects of trust-increasing psychosocial interventions.

## Introduction

The prevention of youth violence represent a significant public concern worldwide^1^. The cost of juvenile antisocial behaviour is known to be high, particularly for those youth who become persistent offenders. In the 5th edition of the American Psychiatric Association's Diagnostic and Statistical Manual of Mental Disorders (DSM-5) and the 10th/11th revision of the International Classification of Diseases (ICD-10/-11) antisocial and violent behaviours in youth is addressed by the diagnosis of conduct disorder (CD). A major criticism of the diagnostic criteria for CD is, that they are based on behavioural symptoms and not on the underlying cognitive or emotional processes that drive these symptoms^2,3^. Now, DSM-5 and the upcoming ICD-11 further allow to differ between subtypes of CD according to the presence of “limited prosocial emotions” (referring to callous-unemotional traits that includes a lack of empathy, a lack of remorse, shallow affect, or a lack of concern about performance). A large body of research has investigated callous-unemotional traits, which were found to go along with a severe, violent, and persistent pattern of antisocial behavior in children and adolescents^3,4^.

Despite youth specialist forensic services having been introduced in many countries, and effective interventions for antisocial youth being available, the treatment of juvenile offenders remains a challenge for many child- and adolescent-psychologists and psychiatrists. The identification of additional useful treatment approaches for changing delinquent behaviours/attitudes in order to impede criminal recidivism is necessary.

Interpersonal trust has been described as a core dimension of cooperative, mutually beneficial interpersonal relationships and the basis for the development of a concept of fairness and moral identity in children and adolescents^5,6^. Trust can be defined as “a psychological state comprising the intention to accept vulnerability based upon the positive expectations of the intentions or behaviour of another”^7^^,p. 395^. Interpersonal trust portrays a social dilemma, because trusting another person is always associated with the risk of being cheated or exploited. Although high levels of social trust seem beneficial for an individual, it would be a failure to trust anybody.

From a developmental perspective, interpersonal trust arises from the child’s attachment to caregivers in early childhood. According to Erikson’s psychosocial theory^8^ children gain a sense of trust that the world is good during their first year of life as they receive warm and responsive care. Children develop an “inner working model” on trustful or unreliable relations based on the experiences of early interactions with primary caregivers^9^. In line with these considerations, social trust was found to increase with age during childhood and adolescence^10^. Unsecure bonding, inconsistent and harsh parenting and/or early child maltreatment affects the ability to develop future trustful relationships to others and increase the risk of psychiatric disorders^5,11^. Accordingly, low trust was found to be associated with schizophrenia, borderline personality disorder and post-traumatic stress disorders^12–14^.

From a theoretical perspective, low interpersonal trust is also related to juvenile offending, CD, and CD-associated callous-unemotional traits: First, the experience of early trustful relations to primary caregivers is an important basis for the moral development of children and adolescents^5^. Moral deficits were found to be consistently related to antisocial behaviours and criminal recidivism in youths and adults^15,16^. Second, childhood adversity and conduct problems were found to impact neural development for the recognition of others’ emotions (see research on amygdala and responses to facial expression of anger)^17–19^ and the hostile attribution of others’ intentions in social interactions was found to drive aggressive behaviour^20,21^. Third, the ability to build trustful social relationships is an important factor for stimulating cognitive and affective empathy^22^.

Aligning with the recent move towards using Research Domain Criteria (RDoC) that aim to provide a biological framework for understanding mental disorder (based on direct observations rather than just on reports of symptoms; see e.g., Fonagy and Luyten^23^), neuroeconomic games such as the “Trust Game” (TG) seem to be a good paradigm to measure interpersonal trust^24^. One player (the investor) is endowed with a certain amount of money (or points that represent a specific monetary value). The investor can decide whether to keep all the money or to “invest” and to transfer some amount to his partner in the TG (the trustee). The amount invested is tripled in value and sent to the trustee, who has to decide what proportion to return to the investor. Among children and adolescents from community samples, two previous studies found a negative association between externalizing behaviours (conduct and oppositional defiant problems) and investments in a TG^25,26^. However, a more recent study in a sample of psychiatric inpatients found youth with externalizing behaviour problems invested less in the first rounds of a repeated TG^27^. Among male adults, one study reported lower investments in adult offenders under community supervision compared to non-delinquent controls^28^. Furthermore, outcomes of a TG depend on the opponent’s characteristics: lower investments were found in TGs against human opponents only and were not reported in TGs against a computer opponent^29^. The heterogeneity of the samples and a broader definition of externalizing behaviours may be responsible for the inconsistency of previous findings.

CD related callous-unemotional-traits seem to result from interpersonal deficits in early childhood^23^ but their relation to interpersonal trust is unclear. White et al.^30^ found fear-specific emotion recognition deficits in children with high levels of callous-unemotional-traits. Such neurophysiological deficits may lead to poorer and less successful social interactions with others and possibly results in lower levels of social trust. In addition, although several risk assessment instruments exist for predicting criminal recidivism in youth^31^, and socio-emotional deficits were considered as risk factors in some of them (e.g., Psychopathy Checklist Youth Version)^32^, no previous study so far has tested the association of interpersonal trust with any criminal and/or violent re-offenses.

Building on previous research on neuro-economics and psychiatric disorders^33–35^, the present study aimed at analysing a subsample of male juveniles who committed serious violent offenses and met criteria of conduct disorder (JO/CD) and a subsample of healthy controls (HC) using a series of one-shot trust games (TGs) against different unknown human opponents or against a computer. Four principal research questions were examined in this exploratory study: (1) do JO/CD show lower investments than HC? (2) Do investments of human vs. computer condition differ in TGs? (3) Is overall investment in TGs correlated with callous-unemotional traits in JO/CD? 4) Is overall investment in TGs associated with any future criminal or violent re-offenses in JO/CD?

## Results

### Clinical characteristics of the samples

Information on study participants (e.g., age, foreign nationality, living situation), current psychiatric disorders and ICU scores of the total sample and the JO/CD and the HC subsamples is shown in Table 1. Out of 24 JO/CD, 5 were on psychiatric medication. Two participants with attention-deficit-hyperactivity disorder were medicated at the time of the assessment. According to clinical impression and the self-declaration of the participants, none of them was under alcohol or drugs at the time of the experiments”.

**Table 1:** Characteristics of study participants (at baseline), current psychiatric disorders, and callous unemotional traits of the total sample (N = 48), the JO/CD subsample (n = 24), and the HC subsample (n = 24).

Variables	Total sample	JO/CD (n = 24)	HC (n = 24)	Test statisics^a^	p value
**Characteristics of study participants**					
Age (years) (mean, *SD*)	17.81 (1.50)	17.71 (1.60)	17.92 (1.41)	-0.48	.635
Foreign nationality (*n*, %)	10 (20.8%)	5 (20.8%)	5 (20.8%)	0.00	1.00
Living at home with both parents	23 (47.9%)	6 (25.0%)	18 (75.0%)	10.10	.001
Out of home placement	7 (14.6%)	5 (20.8%)	2 (8.3%)	1.51	.220
School problems (previous class repetition)	15 (31.3%)	13 (54.2%)	2 (8.3%)	–	.001
School problems (any truancy in the last year)	13 (27.1%)	9 (37.5%)	4 (16.7%)	–	1.93
**Current psychiatric disorders**					
CD	24 (50.0%)	24 (100.0%)	0 (0.0%)	1.00	.000
Substance related disorder	10 (20.8%)	10 (41.7%)	0 (0.0%)	16.63	.001
Affective disorder	3 (6.3%)	3 (12.5%)	0 (0.0%)	3.20	.234
Anxiety disorder	3 (6.3%)	3 (12.5%)	0 (0.0%)	3.20	.234
ADHD	2 (4.2%)	2 (8.3%)	0 (0.0%)	2.09	.489
Any psychiatric diagnosis other than CD	15 (31.3%)	15 (62.5%)	0 (0.0%)	21.82	.000
**Callous-unemotional traits**					
ICU total score (mean, *SD*)	24.08 (8.41)	27.58 (8.91)	20.58 (6.29)	3.14	.003
ICU callous (mean, *SD*)	8.17 (4.36)	9.92 (4.92)	6.42 (2.89)	3.01	.004
ICU uncaring (mean, *SD*)	8.54 (3.77)	9.88 (3.40)	7.21 (3.71)	2.60	.013
ICU unemotional (mean, *SD*)	7.38 (2.34)	7.79 (2.23)	6.96 (2.42)	1.24	.221

*Note*: JO/CD = male juveniles who committed serious violent offenses and met criteria of conduct disorder, HC = Healthy controls, SD = standard deviation, CD = Conduct disorder, ADHD = Attention-Deficit-Hyperactivity-Disorder, ICU = Inventory of Callous-Unemotional Traits, ^a^t-test or χ^2^-test/Fishers’s exact test.

### Findings of one-shot TGs

**Figure 1:**
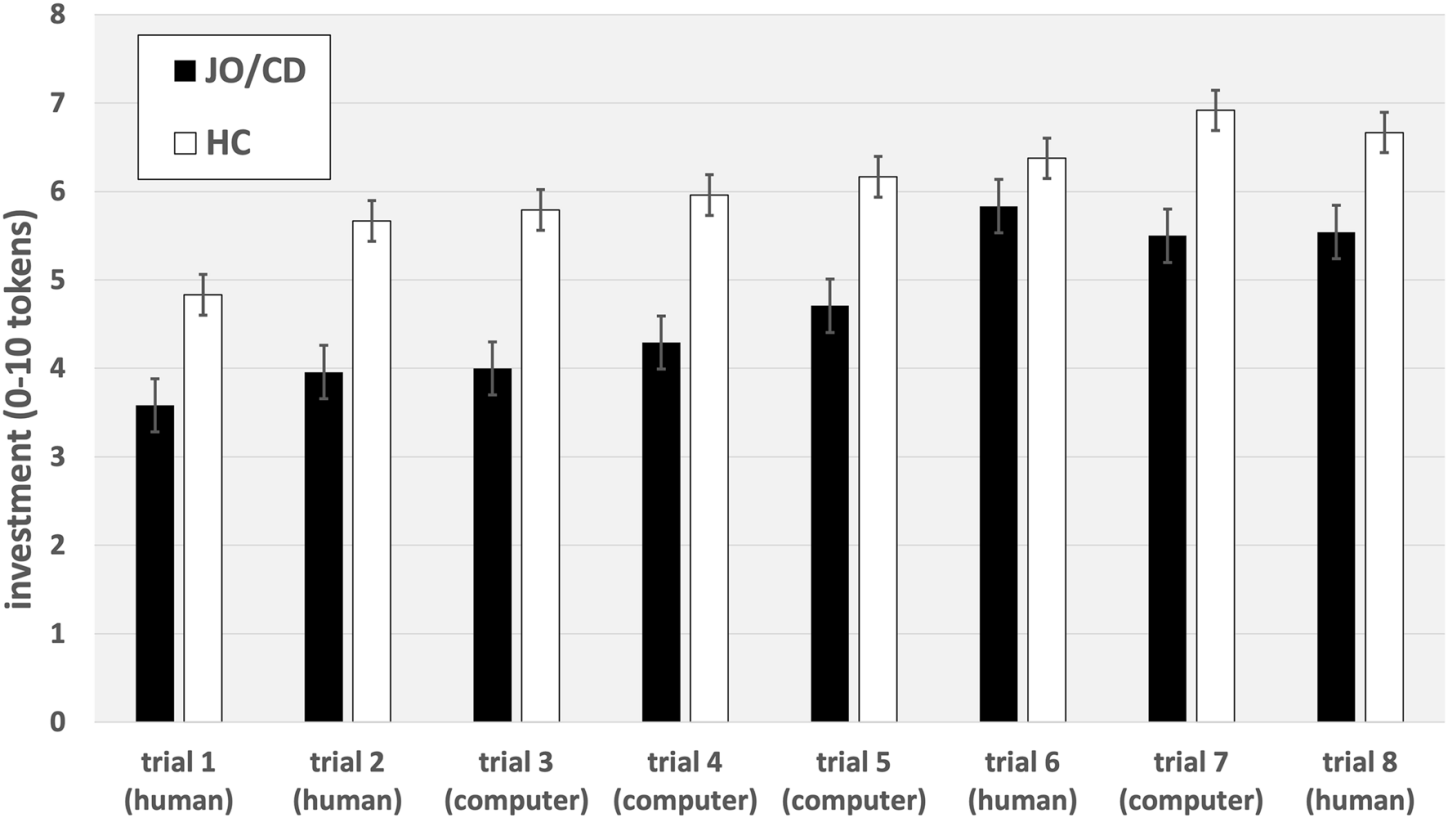
Mean investments and standardized errors in TGs 1–8 of JO/CD and HC.

Mean investment (of possible 0–10 points) in the first trial against a human was 4.21 points (SD 2.41 points) for the complete sample, 4.82 points (SD 2.43 points) for HC, and 3.48 points (SD 2.26 points) for JO/CD. Mean investment in the first trial against a computer was 4.90 points (SD 3.09 points) for the complete sample, 5.79 points (SD 3.15 points) for HC, and 4.00 points (SD 2.81 points) for JO/CD. Mean overall investment of all eight rounds in TGs was 42.89 points (SD 20.06 points) for the complete sample, 48.37 points (SD 19.93 points) for HC, and 37.42 points (SD 19.04 points) for JO/CD. Figure 1 shows mean investments in trials 1–8 for JO/CD and HC, separately.

The findings of the four tested mixed effect models (with and without interaction terms and with and without considering a random slope of condition in participants) with subjects as random factor and group and condition as fixed factors are presented in Table 2. Based on likelihood ratio tests, the addition of an interaction term (model 2), random slopes for condition (model 3) or both (model 4) did not improve the model fit (compared to model 1). According to the AIC model 3 and according to BIC model 1 was found to best fitting our data. Based on these findings and by considering the interpretability, we decided to use model 1 as the most simple solution.

**Table 2:** Findings from mixed effect models with group and condition as fixed factors and subjects as random factors on investments.

	Model 1 (including random intercept)	Model 2 (including random intercept)	Model 3 (including random intercept and random slope)^a^	Model 4 (including random intercept and random slope)^a^
	*S* (95% CI), *p* value	*S* (95% CI), *p* value	*S* (95% CI), *p* value	*S* (95% CI), *p* value
**Fixed effects**				
Group (JO/CD = 1, HC = 0)	− 0.44 (− 0.88–0.01), *p* = .054	− 0.51 (− 0.97–0.04), *p* = .034)	− 0.39 (− 0.83–0.04), *p* = .075	− 0.51 (− 1.01–0.01), *p* = .049
Condition (human = 1, computer = 0)	− 0.03 (− 0.17–0.10), *p* = .599	− 0.10 (− 0.29–0.08), *p* = .273	− 0.03 (− 0.19–0.12), *p* = .650	− 0.10 (− 0.31–0.11), *p* = .341
Group × condition	−	0.14 (− 0.12–0.40), *p* = .305		0.13 (− 0.16–0.44), *p* = .373
**Covariates**				
Age	− 0.01 (− 0.23–0.21), *p* = .934	− 0.01 (− 0.23–0.21), *p* = .934	0.01 (− 0.21–0.22), *p* = .956	0.01 (− 0.21–0.22), *p* = .956
**Model parameters**				
2-log likelihood	− 873.685	− 873.150	− 871.194	− 870.749
AIC	1759.369	1760.301	1758.298	1759.498
BIC	1783.073	1787.955	1789.903	1795.054

^a^Including random slope on condition (human = 1, computer = 0), *JO/CD* male juveniles who committed serious violent offenses and met criteria of conduct disorder, *HC* healthy controls, *AIC* akaike information criterion, *BIC* Bayesian information criterion, *S* standardized coefficient, *CI* confidence interval.

JO/CD invested 0.44 (95% CI − 0.01 to 0.89) CHF (almost 10% of their seed capital) less per round on average than HC, although the effect was not statistically significant (p = 0.054). In contrast, no effect was found for the human vs. the computer condition. Age was not found to have a significant effect on investment. Additional analyses including trial number as additional factor in mixed models 5–8 (supplemental table S1) suggest that model 7 (without interaction term and including a random intercept and random slope of condition) most accurately fit the data. In this model, and similar to the main results above, JO/CD showed a non-significant trend to invest less than HC and no differences were found for human vs. the computer condition of TGs. However, the significant effect of trial number suggests a small increase in investment over time (0.09 CHF per round, 95% CI 0.07–0.12, p < 0.001).

### Association of overall investment in TGs with self-reported callous-unemotional-traits

The Spearman correlation matrix of overall investment in TGs, the ICU total score and subscales ICU-callous, ICU-uncaring, and ICU-unemotional for JO/CD is shown in Table 3. For the ICU total score, no significant correlation was found between overall investment in TGs. For the subscales a significant negative correlation was found between overall investment in TGs and the ICU-uncaring but not between overall investment in TGs and ICU-callous or ICU-unemotional. In further exploratory analyses of the subsample of JO/CD no significant association was found between overall investment in TGs and any comorbid psychiatric disorders (r = − 0.082, p = 0.703) and any substance use disorders (r = − 0.209, p = 0.326).

**Table 3:** Spearman correlations of the overall investment in TG, ICU total score and ICU subscale in JO/CD.

Measures	1	2	3	4	5
1 overall investment in TG	–				
2 ICU total score	− .332 (p = .113)	–			
3 ICU callous	− .230 (p = .279)	.938 (p < .001)	-		
4 ICU uncaring	− .428 (p = .037)	.842 (p < .001)	.706 (p < .001)	–	
5 ICU unemotional	− .123 (p = .566)	.692 (p < .001)	.540 (p = .006)	.398 (p = .054)	–

JO/CD = male juveniles who committed serious violent offenses and met criteria of conduct disorder.

### Criminal recidivism

Of the 24 in the JO/CD subsample, 15 (62.5%) committed one or more criminal re-offenses and 7 (29.2%) committed one or more violent re-offenses. The proportional hazard assumption was not found to be violated for overall investment in TGs and age (p > 0.5). Findings of univariate und multiple Cox-regression with overall investment of TGs as predictor and time to first violent or general re-offense as outcome variables are shown in Table 4. Model 2 further includes age as covariate in the analyses. Hazard ratios (HRs) indicate a significant negative association of the overall investment in TGs with later general re-offenses but no association with later violent re-offenses. JO/CD with smaller overall investments in TGs committed general re-offenses more frequently in a shorter time period after the TGs. This association was still confirmed when age was included as covariate in the analyses.

**Table 4:** Findings from univariate Cox-regressions with overall investments in TGs as predictors of violent recidivism and general recidivism in JO/CD subsample (n = 24).

Predictors	*Any criminal recidivism*		*Any violent recidivism*	
	*HR (95% CI)*	*p value)*	*HR (95% CI)*	*p value*
**Model 1**				
Overall investment in TGs (z-transformed)	0.49 (0.24–0.99)	.048	1.09 (0.51–2.36)	.820
**Model 2**				
Overall investment in TGs (z-transformed)	0.41 (0.21–0.83)	.014	0.92 (0.42–2.03)	.838
Age	0.76 (0.54–1.05)	.096	0.76 (0.48–1.22)	.255

*Note:* male juveniles who committed serious violent offenses and met criteria of conduct disorder, HR = Hazard Ratio.

## Discussion

The main aim of this study was to examine if JO/CD show lower investments than HC in a series of one-shot TGs. Mixed model analyses revealed no statistically significant effect between groups (using two tailed testing). Despite the statistically non-significant result, our data suggest a trend that JO/CD from the forensic outpatient treatment sample invested less points than HC during TGs, but our sample was too small to detect it (by interpreting the point estimate of − 0.44 with 95% confidence intervals of − 0.88 to 0.01, p = 0.054). This result was also confirmed by additional analyses including trial number as an additional factor in the analyses. Possible deficits in social trust should be addressed by further research including larger samples and more juveniles with more severe offenses (e.g., samples from residential care or juvenile detention facilities). Although we included age as a covariate in the analyses, which was unrelated to investment, CD may behave differently in younger compared to older aged adolescents.

Although, the group effect closely missed significance our findings mirror previous findings of adults who were under supervision because of criminal offenses and were found to invest less in a one shot TG against an unknown opponent than community controls^28^. However, in contrast to the study of Clark, et al.^28^, we examined a series of one-shot TGs involving different human and computer opponents and found consistently lower investments in JO/CD for each trial. Previous findings only partly confirm trust deficits at the beginning of an interaction with specific human opponents in youths^25–27^. Their findings were based on a broader definition of externalizing behaviours and the inclusion of younger children of both genders.

Juvenile antisocial behaviours with lower social investments in interactions seem to reflect a general social mistrust and a “hostility bias”^20^ that may go along with previous and current social experiences of adolescents and young adults with antisocial behaviours. Offenders tend to believe in an unjust world^36^. A number of studies have found an accumulation of adverse childhood experiences (ACE; including e.g., physical childhood maltreatment, neglect, parental separation) to be strongly associated with the development of CD and current/future violent behaviours in adolescence and young adulthood^37,38^. In line with this research, a recent study among incarcerated males found interpersonal trust deficits as a consequence of an inability to form moral impression on harmfulness of others after the exposure to violence^39^. However, although the general strategy used by the JO/CD is not the optimal strategy under trustful conditions, it may be the optimal strategy given the rules of a criminal and egoistic world. In summary, our findings suggest that social trust is an interesting concept that should be addressed further to understand the development of aggressive and violent behaviours.

Our second research question relates to additional effects of human vs. computer condition on investments in TGs. Investments in TGs did not differ between the human and computer conditions. Previous studies suggest that trust deficits were specific to social interactions with humans^27^. In a lottery condition where outcomes were randomly selected by a computer algorithm from a range of possible outcomes no differences were found between youth with externalizing behaviour problems and controls^27^. Such a lottery setting is particularly related to risk taking decisions that were found to be higher in children and adolescents with ADHD^40^, that is also frequently comorbid with CD^2^. However, the computer condition in the current study is more comparable with a real social interaction because the algorithm randomly selected a previous response from a database of human opponents (in order to warrant similar returns and to produce realistic responses). Therefore, the fact alone that the participants knew that a computer algorithm makes the decision seems not to be relevant for decision making. Probably, the participants expectations of higher returns by computers becomes of importance in lottery games.

In additional analyses including trial number as predictor in mixed models (see supplemental table S1) we found a significant effect of time course of trials. This finding was rather unexpected as in the present study participants played TGs against different unknown human opponents and it was not possible to learn from previous interactions. Our findings mirror studies of repeated TGs that were played against the same human opponent. In such settings trust emerges as the outcome of a reciprocal social relationship^41^. We have no final explanation for this finding. It can be assumed that trust may also arise from unspecific experiences of social success or that some youth behave like they were playing a repeated TG even they know that this was not the case. The increase of investments in JO/CD further suggest that also antisocial youths are able to increase social trust but may need more time in a trustful environment than healthy subjects. This may be important for interventions with JO/CD.

Further analyses in the subsample of JO/CD focused on the association of trust with self-reported callous-unemotional traits. No association of the ICU total score as well as of the ICU-callousness and ICU-unemotional with overall investments was found in JO/CD. Hence, our findings do not support the idea that general callous-unemotional traits or more specific emotional dysfunctions such as a lack of empathy and remorse or emotional perception were relevant for JO/CD’s performance in one-shot TGs. However, a significant negative correlation of − 0.428 between the ICU-uncaring and overall investment in TGs in JO/CD was detected. JO/CD with higher investments were found to be less uncaring about others. This finding supports the relevance of trust related abnormalities in the moral development in some adolescents with CD^5^ that may linked to early deficits in social bonding to caregivers^42^. Further exploratory analyses revealed no association of total investment in TGs with any comorbid psychiatric disorders/comorbid substance use disorders. Substance use disorders are well-known comorbidities of CD^43^ and highly prevalent in juvenile detention samples^44^.

Finally, we tested trust as predictor of criminal recidivism. Findings from univariate Cox-regressions revealed that overall investment in TGs is a significant predictor of general but not violent recidivism. The more JO/CD invested in TGs, the lower was the risk for committing any further offense within the next 2.9–7 years. Although this finding is based on a small sample of 24 JO/CD, it suggests a potential for TG as a prognostic tool in forensic psychiatry/psychology. Interpersonal trust seems not only related to current antisocial behaviours but also to have long lasting effects on criminal behaviours. This result further supports the concept of interpersonal trust to understand social behaviour in adolescents and young adults charged or convicted for violent offenses. To the best of our knowledge, this study is the first study to examine the effects of investments in TGs on criminal recidivism. Future studies may address this issue within larger samples of JO/CD subjects, controlling for well-established prognostic factors such as criminal history, psychopathology and personality traits.

The limitations of the study include the following: the current findings are based on a rather small sample of one forensic institution and aged matched controls. The small sample size and the focus on males limit the generalizability of the findings. Further replications in severely offending juveniles (e.g., from residential care, juvenile detention centres) and larger samples seem necessary. Although HC were interviewed to exclude psychiatric disorders and (serious) criminal activities no further validation of their declarations was possible. No information on participants socioeconomic background and on adverse childhood experiences was available. Both were previously reported as a risk factor for criminal offenses and recidivism^37,45,46^. Furthermore, no information on the severity, the beginning and the duration of CD and the presence of antisocial personality disorder (for JO/CD older than 18 years) was available. Although IQ < 80 was considered as exclusion criteria in both subsamples, we cannot rule out that IQ and other factors may affected the findings of the TGs. The definition of trust as investment in TGs may differ from trust-based decisions in daily life of adolescents. Future studies may benefit from the inclusion of a questionnaire addressing relational trust. Furthermore, the limited statistical power impeded the performance of further analyses on covariates. Considering only official reoffending data, we cannot rule out that some re-offenses may not have been reported to the police or were not charged by justice authorities.

The current finding on social trust deficits in CD suggests that trust may be an interesting concept for forensic psychiatry and may impact further research. In addition, it suggests that measuring social behaviours in real time during game play may offer new possibilities for forensic assessments. Given the tendencies of juveniles to deny or minimize problem behaviours in questionnaires and interviews, behavioural economic approaches may serve as additional possibilities to study underlying mechanisms of CD symptoms, to estimate the risk of further offending and to tailor interventions. The use of self-report questionnaires to assess externalizing behaviours is limited, because they refer to the participants general knowledge on socially acceptable behaviours rather than revealing the participants actual feelings and behaviours during social interactions. Our results suggest that interpersonal trust may help to understand antisocial behaviours in individuals with JO/CD and may be used to predict general criminal recidivism. In addition, our findings of increasing trust in JO/CD individuals during TGs 1–8 suggests trust may represent a target for therapeutic intervention for CD.

## Methods

### Participants

#### Juveniles who committed serious violent offenses and met criteria of conduct disorder (JO/CD)

Data were collected between October 2013 and December 2017 from male adolescents referred by juvenile justice authorities for a forensic treatment at the Child and Adolescent Forensic Outpatient Unit of the University Hospital of Psychiatry Zurich. Inclusion criteria were the presence of a conduct disorder (CD; according to MINI-KID criteria see below), male gender, age of 14–21 years, and the willingness of the participation in the current study provided by an informed consent of the youth and a parent (for adolescents below age of 18 years). Exclusion criteria were any current psychotic disorder, any current substance dependence, any current or past severe eating disorder, any current depressive or manic episode and a severe attention-deficit disorder (according to MINI-KID and clinical impression of the therapists) as well as an IQ < 80 (according to clinical impression). The final JO/CD sample consisted of 24 male participants (mean age 17.71 years, SD 1.60 years, range 14–20 years). All of them were adjudicated or convicted of having committed serious violent offenses according to the Swiss penal law (e.g., violent assault, robbery, sexual offenses). Eligible probands were approached by their therapists. Subsequently, they have been invited personally to participate in the study by the first author (MA) who worked at the same unit and joined at the beginning of a therapy session. After agreeing to participate, further appointments were arranged with the independent experimenter (MH). Confidentiality was assured for all information that was collected in the study.

#### Healthy controls (HC)

Data were collected between January 2014 and October 2017 on male adolescents recruited via flyers in schools. After responding they were directly invited by the experimenter (MH). Inclusion criteria were comparable to JO/CD. Exclusion criteria were the presence of any current psychiatric disorder (according to MINI-KID including CD), an IQ < 80 (according to clinical impression) and the presence of criminal activities or convictions (according to interviews with HC). The final HC sample was matched on age and consisted of 24 male participants (mean age 17.92 years, SD 1.41 years, range 16–21 years).

### Measures

#### Conduct disorder (CD)/psychiatric disorders

The presence of a psychiatric disorder (including CD and comorbidity) was assessed via the Mini-International Neuropsychiatric Interview for Children and Adolescents (MINI-KID), a short structured clinical diagnostic interview designed to assess the presence of psychiatric disorders in children and adolescents^47^. Diagnoses were based on an algorithm that is appropriate for symptom count, age, duration, and impairment according to DSM-IV criteria. We assessed the presence of conduct disorders (CD; inclusion criteria for JO/CD) as well as substance related disorders, affective disorders (e.g., major depression, dysthymia), anxiety disorders (e.g., social phobia, separation anxiety, panic disorders), and attention-deficit-hyperactivity-disorder. The MINI-KID has been shown to have good reliability and validity when compared with the Schedule for Affective Disorders and Schizophrenia for School-Aged Children—Present and Lifetime Version^47^.

#### Callous-unemotional traits

The self-report version of the Inventory of Callous-Unemotional traits [ICU^48^] was used as a dimensional measure to study callous-unemotional traits in JO/CD and HC. The scale consists of 24 items using a 4-point Likert scale from 0 (not at all true) to 3 (absolutely true). Factor analyses confirmed a general ICU total score and three distinct factors: callousness (e.g., “The feelings of others are unimportant to me”), uncaring (e.g., “I try not to hurt others' feelings”, reversed score), and unemotional (e.g., “It is easy for others to tell how I am feeling”, reversed score). The ICU was found to be a reliable and valid measure in adolescents (using the German version of the ICU) and juvenile offenders^49,50^. For the present study the ICU total score and the subscales were used (Cronbach alpha: ICU total score = 0.822, ICU-callousness = 0.730, ICU-uncaring = 0.707, ICU-unemotional = 0.524). No official cut-off scores are available for the ICU. A recent study^51^ analysed possible cut-off scores in a community and a juvenile detention sample from the US and suggested an empirical based cut-off score of 24.

#### Criminal recidivism

Information on criminal recidivism after TGs of the 24 JO/CD (mean time 5.5 years, SD 1.14 years, time range 2.91–6.98 years) were drawn on November 18, 2020 from official records by the Swiss Federal Office of Justice. In line with previous studies [e.g.,^52^] on juvenile offending we first analyzed (time to) any criminal re-offense. In a second step, we analyzed (time to) violent re-offenses as an indicator for more serious offenses. Violent crimes were considered as both general reoffences and violent re-offences. Any criminal recidivism was coded according to any conviction after TGs or any current adjudication on crimes as defined by the Swiss Penal Code. Violent recidivism was coded based on any new conviction or current adjudication on crimes as defined by the section “crimes against body and life” in the Swiss Penal Code (e.g., murder, manslaughter, bodily assault) as well as robbery, coercion, and any contact sexual offenses (rape, sexual coercion). Besides the presence of any new (violent) criminal conviction or adjudication we also coded time to the first re-offense and first violent re-offense after the assessment based on the offending dates reported in files.

### Procedure

#### One-shot trust games

Each participant played a series of eight one-shot trust games (TGs). The participants acted as investors against a recipient/trustee, either a human player (trial 1, 2, 6, and 8) or a computer (trial 3, 4, 5, and 7). In each TG, players have ten points as seed capital (each point represents a value of 0.5 Swiss francs). The investor is now asked to transfer an amount × ranging from 0 to 10 points from their capital to the recipient. This amount is tripled, common for trust games (***Camerer, 2003). The recipient/trustee can now decide, how many points he wants to return to the initial investor (ranging from 0 points to the possible maximum of his points available). The participants were immediately informed of the amount that was returned from the trustees.

In the human condition, the role of the opposing player was played by the investigator (MH), using the responses of trustees who had played previously. Participants randomly choose one opponent for each trial out of a pool of n = 76 youths who have played the game previously recording all their possible reactions based on the prior investment from 0 to 10. In the computer condition participants were informed that the amount returned from the trustee was based on the decision of a computer algorithm randomly selecting from all previous trustee responses. Before the beginning of the testing, TGs were carefully explained to all participants and examples of a TG against a human player and of a TG against a computer were performed to see if they understood the procedures. In the human player condition, it was clear for participants that the decisions were made by an adolescent who had played the game before. After completing the TGs, one trial of the human condition (round 1, 2, 6, or 8) was randomly selected for pay off.

#### Statistical analyses

Mixed models were used to analyse main and interaction effects of group (JO/CD vs. HC) and condition (human vs. computer) on investments in one shot TGs (trial 1–8). Analyses were performed in R Statistical Software (version 3.6.3) using packages “nlme”^53^ using maximum likelihood estimations. We fit four models to the investment data. Model 1 contained group and condition as independent factors. Model 2 added the interaction between group and condition. To control for interindividual variations, subjects were defined as a random factor (by including a random intercept in the analyses) in all models. In models 3 (without interaction) and 4 (with interaction), we additionally considered a random slope on condition (human vs. computer). Age was considered as covariate in all analyses. In order to choose a single model to evaluate, we used likelihood ratio testing for nested models, the Akaike Information Criterion [AIC^54^] and the Bayesian Information Criterion [BIC^55^], where lower AIC or BIC values indicate a better fit to the data. Although we did not use a repeated trust game, where participants played several rounds against the same opponent, we cannot exclude the presence of a time effect between trials 1–8. To test this, we repeated the mixed model analyses that we performed before by including trial number as an additional independent factor in the analysis. For all mixed models, standardized coefficients and 95% confidence intervals were included to compare effects (see discussion on effect sizes in mixed models)^56^.

Spearman correlations were used to analyse associations of overall investment in TGs and ICU total score and subscales in JO/CD. Cox-regressions were used to analyse the association of overall investment in TGs with time to any criminal recidivism and time to any violent re-offenses. The Cox-proportional hazard model is essentially a regression model commonly used in medical research for investigating the association between the survival time of patients and one or more predictor variables. In the present study, the time to the first criminal re-offense was considered as survival time (dependent variable) and overall investment in TGs as independent variable (model 1). Based on the well-known association of young age and increased risk for criminal (re-)offenses^57,58^, age was as included as a covariate (model 2). The hazard ratio from Cox regression can be interpreted as relative instantaneous risk^59^. It indicates the change in the risk of an event (e.g., a criminal re-offense) if the parameter in question (e.g., overall investment in TGs) rises by one unit. A hazard ratio of one means lack of association, a hazard ratio greater than one suggests an increased risk, and a hazard ratio below one suggests a smaller risk. Statistically significant hazard ratios cannot include unity (one) in their confidence intervals. The proportional hazard assumption (it essentially means is that the ratio of the hazards for any two individuals is constant over time) was tested for overall in TGs and age.

Analyses were performed in SPSS (version 24) and R statistic software version 3.6.3^60^. The level of significance was set to be p < 0.05 (two tailed).

### Ethics approval

Ethical approval was granted by the ethics committee of the Canton Zurich (Ref. no. 2012-0438). This study was performed in accordance with the ethical standards as laid down in the 1964 Declaration of Helsinki and its later amendments or comparable ethical standards.

### Informed consent

Informed consent was obtained from all participants and a legal guardian (for adolescents below age of 18 years).

## Supplementary Information

Supplementary Information.

## Data Availability

Data are available on request by the corresponding author.
