# Supplementary material for: Associations of interpersonal trust with juvenile offending/conduct disorder, callous-unemotional traits, and criminal recidivism
Source: Sci Rep. 2022 May 9;12:7594. doi: 10.1038/s41598-022-11777-6 (PMC9085823; doi:10.1038/s41598-022-11777-6)
Supplement: Supplementary file 1 — Supplementary Information. [file 41598_2022_11777_MOESM1_ESM.pdf]

**Supplementary information:**

# Associations of interpersonal trust with juvenile offending / conduct disorder, callous-unemotional traits, and criminal recidivism

Marcel Aebi*[1,2], ORCID: 0000-0001-7901-9801
Melanie Haynes[,3], ORCID: 0000-0002-6525-6838
Cornelia Bessler[1,2]
Gregor Hasler[4], ORCID: 0000-0002-8311-0138

[1]Research & Development, Corrections and Rehabilitation, Department of Justice and Home Affairs, Canton of Zurich, Switzerland
[2]Department of Forensic Psychiatry, University Hospital of Psychiatry Zurich / University of Zurich, Switzerland
[3]Translational Research Center, University Hospital of Psychiatry and Psychotherapy Bern, Switzerland
[4]Unit of Psychiatry Research, University of Fribourg, Fribourg, Switzerland

**Supplemental Table S1.** Findings from mixed effect models with group, condition, and trial 1-8 as fixed factors and subjects as random factors on investments.

| | Model 5 (Including random intercept) | Model 6 (Including random intercept) | Model 7 (Including random intercept and random slope)[1] | Model 8 (Including random intercept and random slope)[1] |
|---|---|---|---|---|
| | *S* (95% CI), *p* value | *S* (95% CI), *p* value | *S* (95% CI), *p* value | *S* (95% CI), *p* value |
| Fixed effects | | | | |
| Group (JO/CD=1, HC=0) | -0.44 (-0.88-0.05), $p$ = .054 | -0.51 (-0.97--0.05), $p$ = .033 | -0.39 (-0.83-0.04), $p$ = .076 | -0.51 (-1.0--0.01), $p$ = .049 |
| Condition (human=1, computer=0) | 0.01 (-0.11-0.13), $p$ = .859 | -0.06 (-0.23-0.12), $p$ = .519 | 0.01 (-0.14-0.16), $p$ = .885 | -0.06 (-0.27-0.15), $p$ = .341 |
| Trial 1-8 | 0.09 (0.07-0.12), $p$ < .001 | 0.09 (0.07-0.12), $p$ < .001 | 0.09 (0.07-0.12), $p$ < .001 | 0.09 (0.07-0.12), $p$ < .001 |
| Group X Condition | - | 0.13 (-0.11-0.38), $p$ = .274 | | 0.14 (-0.16-0.44), $p$ = .373 |
| Covariates | | | | |
| Age | -0.01 (-0.23-0.21), $p$=.934 | -0.01 (-0.23-0.21), $p$=.934 | 0.01 (-0.21-0.22), $p$=.956 | 0.01 (-0.21-0.22), $p$=.957 |
| Model Parameters | | | | |
| 2-Log Likelihood[2] | -852.115 [a, b] | -851.508 | -847.889 [a] | -847.490 [b] |
| AIC | 1718.231 | 1719.015 | 1713.779 | 1714.979 |
| BIC | 1745.885 | 1750.621 | 1749.334 | 1754.486 |

*Note*: [1] = including random slope on condition (human=1, computer=0), [2] = Models with the same subscripts (a, b) did significantly differ from each other in likelihood ratio testing, JO/CD= male juveniles who committed serious violent offenses and met criteria of conduct disorder, HC= Healthy Controls, AIC=Akaike information criterion, BIC= Bayesian information criterion, *S* = standardized coefficient, CI = confidence interval.
